# Evaluation of Medical Students’ Satisfaction With Using a Simulation-Based Learning Program as a Method for Clinical Teaching

**DOI:** 10.7759/cureus.59364

**Published:** 2024-04-30

**Authors:** Ahmed H Mujamammi, Sultan A Alqahtani, Feras A Alqaidi, Bandar A Alharbi, Khaled M Alsubaie, Faisal E Alhaisoni, Essa M Sabi

**Affiliations:** 1 College of Medicine, King Saud University, Riyadh, SAU

**Keywords:** clinical years medical students, basic years medical students, preclinical and clinical medical students, simulation program, clinical teaching, medical students, simulation based learning

## Abstract

Objectives

This cross-sectional analytical study aims to evaluate medical students’ awareness and satisfaction regarding the utilization of simulation-based learning (SBL) as a method for clinical teaching at King Saud University (KSU) over the past 12 months. It seeks to understand how such learning methods enhance students’ self-satisfaction and clinical skills, facilitate the application of learned knowledge, and assess the role of instructors in providing ample practice opportunities in the skills laboratory. Furthermore, the study aims to assess the satisfaction levels of students in both preclinical and clinical years regarding the time allocated for skills laboratory sessions and the integration of high-fidelity technology in simulation-based training programs at KSU.

Methods

In this cross-sectional study, a total of 306 male and female medical students from the College of Medicine at KSU participated, comprising 196 preclinical students (first, second, and third years) and 110 clinical students (fourth and fifth years). Quantitative data was collected through a structured questionnaire on a 5-point Likert scale that showed degrees of satisfaction. The satisfaction was measured based on a 5-point Likert scale that shows the degree of satisfaction from (very dissatisfied, dissatisfied, neither dissatisfied nor satisfied, satisfied, and very satisfied), and we calculated the p-value based on an independent t-test, and the percentage represented the percentage of students who chose satisfied and very satisfied.

Results

The results showed overall satisfaction with SBL (mean: 3.98, 71.10%), and it was recognized as a useful and effective method of learning skills. It is reported that it helped the students implement what they learned. At the same time, lower satisfaction was identified in areas with less allocated time for skill labs. Moreover, lack of accessibility and lack of trained staff were reported, and they should be addressed by providing staff with proper training.

Conclusion

The results of the study will help to understand how students’ learning needs should be addressed. Moreover, providing simulation-based training is a pathway compliant with the best educational standards that should be adapted according to each institution’s singularities. Besides offering further results, the study presents suggestions for further research.

## Introduction

In medical education, clinical skills training courses are vital because they allow medical students to master physical examination, medical interviewing, communication skills, and basic clinical procedures [1]. Medical authorities should take care of students and develop and enhance various medical programs. They should identify objectives and use other strategies to build strong approaches to developing high-quality learning materials, whether traditional methods or newer simulation programs [2]. Despite the effectiveness of clinical training programs, which use real patients to provide high-quality clinical teaching, enhance communication skills, and develop skills for suitable procedures in the clinical field, there are many ethical and legal limitations to these programs, and programs can be one of the most important and favorable options to overcome these obstacles to clinical teaching [3].

Simulation-based learning (SBL) programs are an enhanced learning method used by both undergraduate and postgraduate medical students and for clinical teaching. SBL has created many opportunities to learn new skills in the clinical field without involving real patients. In order to be effective and suitable for all students, SBL needs to be smoothly integrated into students’ curricula to have the maximum benefit [1]. SBL can be used to develop many skills and includes many models of mannequins, from simple to computerized full-body mannequins, on which students can test their medical knowledge [1]. Furthermore, this method decreases anxiety and stress while improving knowledge acquisition and memory [4]. Unlike real patients, it is easy to get access to simulators in many clinical settings to provide medical students with a semi-realistic experience [4]. It has been shown that SBL can provide many situations that improve the relationships between real patients and medical students [5,6]. SBL programs are a safe way for students to practice, learn, and communicate effectively without being afraid of harming patients [7]. SBL is valuable and effective in many ways in the medical field and should be integrated as an essential part of medical curricula [8]. It can be used not only by medical students in clinical training but also throughout all medical fields, from college students to many healthcare providers [9]. Although SBL programs are effective and widely used by many medical schools, they are not commonly used in some countries as a method for clinical teaching, such as Japan [10]. SBL for clinical teaching is a valuable method to ensure patients’ safety and decrease the risk of errors when practicing skills such as cardiopulmonary resuscitation, airway management, procedural training, trauma, and risk and crisis management. Our concern is that the effectiveness and value of simulation-based training programs have not yet been proven with enough evidence to support medical students in becoming better doctors with practiced clinical skills compared to classical learning methods [11].

This study evaluates medical students' satisfaction with SBL programs for clinical teaching at King Saud University (KSU) over 12 months in relation to enhancing their self-satisfaction and improving their clinical skills, including whether instructors provided more opportunities for students to practice in skills laboratories.

## Materials and methods

Design

This analytical, cross-sectional study was initiated in June 2021 and continued until 2022 at KSU, Riyadh, Saudi Arabia. The study aimed to evaluate medical students’ satisfaction with the implementation of a simulation learning program as a method for clinical teaching. It targeted both preclinical students (first, second, and third years) and clinical students (fourth and fifth years). Satisfaction levels were assessed using a 5-point Likert scale, which measured the degree of satisfaction.

Inclusion criteria

Medical students enrolled in the study were all undergraduate students, including both male and female groups, in all years, either from preclinical years (first, second, and third years) or clinical years (fourth and fifth years).

Exclusion criteria

We have excluded all graduated medical students and any medical interns, as well as students who have any disabilities that prevent them from attending simulation classes.

Target population

The study sample was calculated based on the medical student population (1,315 total current KSU medical students, both males and females). A sample size formula was used to determine that the required sample size was 306, with 196 medical students in the first group, which included males and females from the preclinical (first, second, and third years), and 110 medical students in the second group, which included both male and female medical students in their clinical (fourth and fifth years). We used a CI of 95% and a 5% margin of error, a 50% sample proportion, and a 45.01 and 54.99 CI.

Data collection

For a better understanding of student satisfaction, a questionnaire developed by Agha et al. [4], which uses a 5-point Likert scale to show degrees of satisfaction, was used to evaluate most of the dimensions in the questionnaire. The questionnaire related to the following two themes: (1) overall satisfaction and (2) challenges related to the SBL. We contacted the authors and received official permission to use the questionnaire. The questionnaire was handled and distributed by the first principal author. It was stratified into two essential groups: 196 in the first group and 110 in the second group. The first group comprised preclinical students, while the second group comprised clinical students. We communicated with medical education staff from the College of Medicine at KSU and requested a list of student IDs, names, and numbers; we randomly picked one student out of four students (group) using a random generator, and we communicated with them via the WhatsApp application.

Due to the COVID-19 pandemic, the questionnaire was distributed online; a link was sent to the participants. The questionnaire started with an introduction and informed consent and was followed by general questions about demographic information (age, gender, and academic year), duration, and exclusion criteria. If a participant met any of the exclusion criteria, then that person was replaced by another individual.

The second part of the questionnaire was about medical students’ satisfaction with the SBL training program and contained many questions about students’ self-satisfaction, clinical skills, whether the program helped them recognize what they had learned, and the role of instructors in providing knowledge and opportunities in the clinical field. The third part of the questionnaire was about challenges in SBL experienced by all student participants (i.e., male, female, preclinical, and clinical). The last part of the questionnaire included questions about the strengths and weaknesses of SBL, such as the time allocated in skills laboratories and the fidelity of the technology used in the program.

Internal reliability was analyzed using Cronbach’s alpha to determine the extent to which the items in our questionnaire were related to each other. Cronbach’s alpha shows internal consistency based on the average inter-item correlation, and the internal reliability for our questionnaire was 0.933, which is excellent.

Statistical analysis

Data were analyzed using IBM SPSS Statistics for Windows, Version 21.0 (Released 2012; IBM Corp., Armonk, NY, USA). Descriptive statistical analysis of the Likert items was conducted by calculating the frequency, mean, and SD. Students’ satisfaction levels were calculated as percentages by combining the frequency of levels of satisfaction (satisfied and very satisfied) for each item in the questionnaire, and the responses on the Likert scale were calculated for each domain (from 1 = very dissatisfied to 5 = very satisfied). An independent t-test was used to find out the differences between preclinical and clinical students. A p-value of ≤0.05 was considered statistically significant, and 95% CIs were used to report the statistical significance and precision of the results.

Ethical considerations

Informed consent was obtained from all participants that indicated the purpose of both the study and the survey; all participants have the right to withdraw at any time from answering the survey; all participants’ confidentiality and anonymity were protected and perceived; and we have not given any rewards or incentives to the participants.

## Results

Table 1 presents the demographic data showing the distribution of medical students among the preclinical years (first, second, and third years) and clinical years (fourth and fifth years), as well as their age and gender.

**Table 1:** Demographic data showing the distribution of medical students between years The ages of the participants ranged between 19 and 26 years, with an average age of 21.1 years. A total of 147 male students from all years represented 47.6% of the total participants, while 162 female medical students represented 52.4% of the total participants.

Gender	Male: 147 (47.6%)			
	Female: 162 (52.4%)			
Academic years’ main categories	All years	Total	Male	Female
Preclinical medical years participant	Year 1	68 (22%)	31	37
	Year 2	56 (18.1%)	27	29
	Year 3	74 (23.9%)	36	38
Clinical medical years participant	Year 4	47 (15.2%)	22	25
	Year 5	64 (20.7%)	31	33

Table 2 outlines the descriptive statistics for the given 14 items. The items are ranked by their mean scores, SD, percentage, and p-value, which measure the relationship between independent variables and dependent variables. The results showed that overall satisfaction scores with SBL were high, with an overall mean of 3.98, an SD of 0.675, and 71.10% satisfaction. Regarding each statement, the results showed that most students (82.75%) considered SBL a useful strategy for learning, with a frequency rating of 4.24 and an SD of 0.807, while 80.3% believed that SBL helped them apply what they had learned and 80% reported that it helped them retain knowledge. The results also revealed that 78.75% of students considered SBL a useful addition to learning, with a high mean score (4.10), and 77.45% reported that it should be included in courses frequently, with a mean score of 4.16, which agrees with the percentage scores. The data showed that 76.8% of subjects were familiar with the concept of SBL and felt comfortable using a simulated environment, and about 73% felt the need for more training sessions with simulators and that simulators provided a semi-realistic experience. Additionally, 64.5% reported that it made the subject more interesting and improved their psychomotor skills. Furthermore, 54.2% claimed that SBL helped them improve their communication skills and develop clinical decision-making skills and that they treated the mannequin as a real patient. We compared the overall differences between the preclinical and clinical students’ satisfaction with SBL, and there were no significant differences between the two groups (p = 0.896). However, we ran an independent t-test to find out if there were significant differences between the two groups for each statement in the table above. The results showed some significant differences, with preclinical students finding the experience more favorable according to the t-test. Students reported that SBL made the subject more interesting (p = 0.001), patient simulators were a useful addition to learning with real patients, they found it difficult to treat the mannequin as a real patient (p = 0.002), SBL helped them retain knowledge (p = 0.005), and they would like more training with simulators (p = 0.006). These findings confirm the first hypothesis that most KSU medical students, both preclinical and clinical, were familiar with and satisfied with the SBL training program over the last 12 months with regard to enhancing their self-satisfaction (51%) and helping them apply what they had learned (67%). Students were also satisfied that instructors provided them with more opportunities to practice in the skills laboratory (86%).

**Table 2:** Comparison of students’ satisfaction with SBL in relation to their clinical years The p-value was calculated based on an independent t-test, and the percentage represented the percentage of students who chose satisfied and very satisfied based on a 5-point Likert scale that shows the degree of satisfaction from (very dissatisfied, dissatisfied, neither dissatisfied nor satisfied, satisfied, and very satisfied). SBL, simulation-based learning

Statement	Preclinical (first, second, and third years)			Clinical (fourth and fifth years)			Overall			p-value
	Mean	SD	%	Mean	SD	%	Mean	SD	%	
Patient simulators are a useful addition to learning with real patience.	4.23	0.843	84.2	3.89	0.932	73.3	4.1	0.889	78.75	0.002
I would like more training with simulators.	4.16	1.01	82.6	3.8	1.13	64.5	4.03	1.07	73.55	0.006
I am familiar with the concept of SBL.	4.03	0.96	70.9	4.19	0.872	82.7	4.08	0.931	76.8	0.138
SBL is a useful learning strategy.	4.32	0.806	85.2	4.1	0.793	80	4.24	0.807	82.75	0.027
SBL made the subject more interesting.	4.15	0.971	78	3.55	1.16	55.5	3.94	1.08	66	0.001
SBL helps me apply what I learned.	4.22	0.971	80.6	4.05	0.975	80	4.16	0.974	80.3	0.144
SBL should be included in the courses frequently.	4.26	0.84	81.2	3.98	0.995	73.7	4.16	0.907	77.45	0.014
SBL helps me retain knowledge.	4.32	0.837	84.7	4.01	0.918	75.4	4.21	0.878	80	0.005
SBL to provide a semi-realistic experience.	4.11	1	76	3.86	1.08	70	4.02	1.03	73	0.45
SBL helps me with my communication skills.	3.59	1.24	55.6	3.34	1.37	52.8	3.5	1.29	54.2	0.12
I feel comfortable in a simulated environment.	4.1	0.952	75	4.06	0.941	78.2	4.09	0.946	76.6	0.7
SBL developed clinical decision-making.	3.72	1.11	59.2	3.46	1.23	54.5	3.63	1.16	56.8	0.067
SBL improves psychomotor skills.	3.95	0.918	66.3	3.73	1.02	62.7	3.87	0.963	64.5	0.066
I found it difficult to treat the mannequin as a real patient.	3.46	1.18	48.5	3.89	1.13	68.2	3.62	1.18	58.35	0.002
Total	4.04	0.974	73.42	3.85	1.039	69.39	3.98	1.007	71.1	

Table 3 shows the challenges associated with SBL. The results showed that most students were satisfied, indicating that they did not face challenges. According to the data, 86.6% (mean: 4.31; SD: 0.751) reported that cooperation between the students is important for practicing in the skills laboratory, and 71.6% (mean: 3.92; SD: 0.993) mentioned that the instructor gave them the opportunity to practice in the skills laboratory. Moreover, 71.4% (mean: 3.94; SD: 0.995) claimed that they were satisfied with the role of the instructor and their level of knowledge in the skills laboratory, and 70.15% (mean: 3.96; SD: 1.00) reported that the skills laboratory facilities were adequate. Additionally, 59.55% reported that the time allocated in the skills laboratory was appropriate, and 54% reported that they were given the opportunity to see high-fidelity simulators. It should be noted that the results did not show any significant differences between the two groups. These findings support our second hypothesis that clinical medical students would be more satisfied with the time allocated in the skills laboratory (84.4%) than preclinical medical students (26.3%). Moreover, our third hypothesis that most clinical medical students would be more satisfied with the use of high-fidelity technology in SBL (78.6%) compared to preclinical students (67.3%) is confirmed.

**Table 3:** Comparison of students’ perceptions toward challenges associated with SBL in relation to their clinical years The p-value was calculated based on an independent t-test, and the percentage represented the percentage of students who chose satisfied and very satisfied based on a 5-point Likert scale that shows the degree of satisfaction from (very dissatisfied, dissatisfied, neither dissatisfied nor satisfied, satisfied, and very satisfied). SBL, simulation-based learning

Statement	Preclinical (first, second, and third years)			Clinical (fourth and fifth years)			Overall			p-value
	Mean	SD	%	Mean	SD	%	Mean	SD	%	
The instructor gave me the opportunity to practice at the skills laboratory.	3.92	1	69.9	3.92	0.974	73.3	3.92	0.993	71.6	0.974
I was given the opportunity to see the high-fidelity simulators.	3.42	1.13	48	3.67	1.03	60	3.51	1.1	54	0.057
I am satisfied with the role of the instructor and his level of knowledge at the skills laboratory.	3.9	1.03	67.4	4.01	0.918	75.4	3.94	0.995	71.4	0.316
The time allocated for the skills lab is appropriate.	3.67	1.09	58.1	3.6	1.19	61	3.6	1.12	59.55	0.559
Cooperation between the students is important for practicing at the skills laboratory.	4.36	0.755	87.7	4.23	0.741	85.5	4.31	0.751	86.6	0.158
The skills laboratory facilities are adequate.	4	1.01	69.4	3.9	0.981	70.9	3.96	1	70.15	0.443
Total	3.88	1.01	66.75	3.89	0.972	71	3.89	0.993	68.88	

Table 4 shows students’ perceptions regarding the strengths of SBL. Regarding exposure to a semi-realistic environment, 64.7% (mean: 3.83; SD: 1.03) reported that SBL was a semi-realistic experience, and 63.15% (mean: 3.72; SD: 1.04) reported that it provided real patient-related information. Besides, 59.15% (mean: 3.66; SD: 1.10) claimed that it helped familiarize them with a hospital environment. The data obtained from the t-test showed that there were slightly significant differences between the two groups, with preclinical students reporting greater satisfaction than clinical students. Concerning the protected learning environment, the results showed that the students were highly satisfied; 87.75% (mean: 4.4; SD: 0.832) of students reported that it was not a threatening environment, 85.55% (mean: 4.39; SD: 0.782) claimed that it helped with patient safety, and 85.45% (mean: 4.32; SD: 0.757) reported that it provided a chance to learn from mistakes. Moreover, when it came to knowledge retention, 78.7% (mean: 4.13; SD: 0.853) of students reported that it improved conceptualization, and 75.7% (mean: 4.05; SD: 0.925) claimed that it helped their recall. Regarding collaboration and communication, 79% (mean: 4.14; SD: 0.932) of students reported that SBL improved interaction and class participation, and 57.9% (mean: 3.74; SD: 1.14) reported that it enhanced their communication skills. Furthermore, regarding opportunities to practice more, 82.85% (mean: 4.23; SD: 0.839) of students claimed that SBL helped them apply their knowledge, and 79.8% (mean: 4.21; SD: 0.911) reported that it offered the opportunity to practice more. Finally, the data showed that 74.77% (mean: 4.07; SD: 0.933) reported that it motivated learning. The t-test showed some significant differences between the two groups in favor of the preclinical group.

**Table 4:** Comparison of students’ perceptions toward the strength of SBL in relation to their clinical years The p-value was calculated based on an independent t-test, and the percentage represented the percentage of students who chose satisfied and very satisfied based on a 5-point Likert scale that shows the degree of satisfaction from (very dissatisfied, dissatisfied, neither dissatisfied nor satisfied, satisfied, and very satisfied). SBL, simulation-based learning

Statement		Preclinical (first, second, and third years)			Clinical (fourth and fifth years)			Overall			p-value
		Mean	SD	%	Mean	SD	%	Mean	SD	%	
Exposure to the semi-realistic environment	Provides real patient-related information	3.83	1.051	65.3	3.54	1.01	61	3.72	1.04	63.15	0.021
	Familiarization with the hospital environment	3.76	1.08	62.8	3.49	1.11	55.5	3.66	1.1	59.15	0.038
	Exposure to semi-realistic experience	3.93	1.03	69.4	3.65	1	60	3.83	1.03	64.7	0.022
Protected learning environment	Chance to learn from own mistakes	4.39	0.739	87.3	4.2	0.775	83.6	4.32	0.757	85.45	0.035
	The non-threatening learning environment for students	4.4	0.898	84.6	4.39	0.705	90.9	4.4	0.832	87.75	0.853
	Patient safety	4.42	0.784	84.7	4.33	0.781	86.4	4.39	0.782	85.55	0.351
Knowledge retention	Easy to recall	4.07	0.944	76	4.02	0.893	75.4	4.05	0.925	75.7	0.651
	Improve conceptualization	4.17	0.879	80.1	4.06	0.804	77.3	4.13	0.853	78.7	0.247
Collaboration and communication	Improve interaction and class participation	4.21	0.952	80.7	4	0.883	77.3	4.14	0.932	79	0.059
	Enhances communication skills	3.87	1.13	62.2	3.52	1.13	53.6	3.74	1.14	57.9	0.011
Opportunity to practice more	Application of knowledge	4.27	0.856	81.1	4.16	0.807	84.6	4.23	0.839	82.85	0.256
	Ability to practice more	4.27	0.907	79.6	4.1	0.912	80	4.21	0.911	79.8	0.138
Simulation	Motivate learning	4.24	0.887	81.2	3.8	1.03	63.6	4.08	0.966	72.4	0.001
Total		4.14	0.935	76.53	3.94	0.913	73	4.07	0.933	74.77	

Table 5 illustrates the weaknesses of SBL. The data revealed that when it came to lack of accessibility, 53.85% (mean: 3.45; SD: 1.25) of students reported that it provided more opportunities to practice on mannequins, and 44.9% (mean: 3.26; SD: 1.24) reported that it was easy to access the simulator, whereas 48.85% (mean: 3.31; SD: 1.26) claimed that they repetitively practiced skills on simulators. The data also showed that when students were asked about insufficient resources and facilities, 66.5% (mean: 3.91; SD: 1.02) reported there was a need for more simulators to match the number of students, and 64.45% (mean: 3.85; SD: 1.05) claimed that there was a need for better-equipped skills laboratories. Regarding group distribution, 77.25% (mean: 4.09; SD: 1.00) recommended dividing students into small groups, whereas 73.65% (mean: 4.01; SD: 0.942) preferred dynamic groups. The data analysis also showed that 44.1% (mean: 3.37; SD: 1.18) of students reported that staff needed more training, and 38.25% (mean: 3.14; SD: 1.26) claimed that most demonstrators were not familiar with using the simulator.

**Table 5:** Comparison of students’ perceptions toward the weaknesses of SBL in relation to their clinical years The p-value was calculated based on an independent t-test, and the percentage represented the percentage of students who chose satisfied and very satisfied based on a 5-point Likert scale that shows the degree of satisfaction from (very dissatisfied, dissatisfied, neither dissatisfied nor satisfied, satisfied, and very satisfied). SBL, simulation-based learning

Statement		Preclinical (first, second, and third years)			Clinical (fourth and fifth years)			Overall			p-value
		Mean	SD	%	Mean	SD	%	Mean	SD	%	
Lack of accessibility	Opportunity to practice more on mannequins	3.43	1.24	50.5	3.48	1.276	57.2	3.45	1.25	53.85	0.775
	Easy access to the simulator	3.27	1.21	43.4	3.24	1.2935	46.4	3.26	1.24	44.9	0.842
	Repetitively practice skills on simulators	3.27	1.24	45.9	3.38	1.3132	51.8	3.31	1.26	48.85	0.469
Insufficient resources and facilities	Need more simulators to match the number of students	4	0.989	68.4	3.76	1.066	64.6	3.91	1.02	66.5	0.052
	Need for well-equipped skills laboratories	3.91	1.04	68.9	3.75	1.0682	60	3.85	1.05	64.45	0.21
Group distribution	Divided students into smaller groups	4.09	1	74.5	4.09	1.0276	80	4.09	1	77.25	0.96
	Group dynamics	3.95	0.97	67.3	4.1	0.8877	80	4.01	0.942	73.65	0.199
Lack of trained staff	Need more trained staff	3.33	1.21	42.8	3.44	1.1381	45.4	3.37	1.18	44.1	0.413
	Most of the demonstrators are not familiar with using the simulator	3.15	1.25	38.3	3.1	1.2875	38.2	3.14	1.26	38.25	0.747
Total		3.6	1.13	55.55	3.59	1.15	58.1	3.6	1.13	56.86	0.518

## Discussion

The benefits associated with simulations in clinical settings have encouraged medical institutions to initiate the use of simulations in medical curricula. The current study evaluates students’ satisfaction with SBL. This study found that most students were in favor of using SBL.

The results of this study showed overall satisfaction with SBL (71.10%; mean: 3.98). These findings agree with the study by Agha et al. [4]., which also indicated overall satisfaction (mean: 7). Moreover, these results concur with the study by Franc-Law et al. [12], where medical students’ overall satisfaction with a simulation-based curriculum was high (8 out of 10 on a Likert scale). Our findings reveal that students considered SBL a useful strategy for learning; SBL helped them apply what they had learned, and SBL assisted them with retaining knowledge. Students found it a useful addition to learning, and they reported that it should be included in curricula. The results also showed that 55% reported that SBL improved their communication skills and clinical decision-making and that they treated the mannequin as a real patient. These are similar to the results reported by Agha et al. [4], Traynor et al. [13], and Ennen and Satin [14], who reported that students retain and gain both knowledge and confidence during simulation sessions for clinical practice as well as improve their communication skills. They also found that SBL has strong educational effects, with particularly large effects in the psychomotor domain. Boet et al. [7] have suggested that interprofessional simulation education can create conditions for an effective collaborative experience. Besides, Nguyen et al. [15]. found that incorporating simulation into resuscitation training sessions provided students with the confidence to manage similar cases in the future. Chakravarthy et al.’s study [16] revealed that medical students’ knowledge, management skills, confidence, and level of satisfaction with the emergency rotation improved significantly with SBL. Steadman et al. [17] found a better transfer of knowledge in SBL students compared to PBL students. Additionally, numerous studies have pointed out that students who were taught using SBL showed high satisfaction levels compared to those who were taught using traditional methods. The other parts of the questionnaire dealt with challenges associated with SBL and the strengths and weaknesses of SBL. Most students reported that cooperation between students was important for practicing in the skills laboratory, so this point should be addressed to achieve maximum benefits. Students also reported that instructors provided opportunities to practice in the skills laboratory. Furthermore, students considered SBL a protected learning environment that allowed them to learn from their mistakes without negatively impacting patient safety. SBL was a non-threatening learning environment that improved confidence and provided opportunities to practice more. These findings agree with those reported by Agha et al. [4].

This study also confirms the results reported by Ennen and Satin [14], who found that the use of simulation can help overcome some limitations related to concerns for patient safety. On the other hand, the questionnaire results revealed important issues related to the weaknesses of SBL, which should be carefully considered and addressed when using SBL to teach clinical skills. Students reported that they preferred to be divided into smaller groups, which is in accordance with the study by Shanks et al. [8], which showed that residents prefer simulator-based procedural teaching in the form of small group sessions. The results also showed that lack of accessibility and lack of trained staff were the main weaknesses of the SBL program, which must be considered for future SBL education programs; similar findings were reported by Agha et al. [14]. In their study, Heitz et al. [18] found that limited faculty time and clerkship hours were barriers associated with this type of teaching and learning. They also found that financial resources, faculty time, and the volume of students were the main obstacles to additional simulation practice for preclinical medical students.

This study also compared the differences between preclinical and clinical students’ satisfaction with SBL, and the results showed that there were significant differences in favor of the preclinical group. These findings support El Naggar and Almaeen’s findings [19], who reported a significant difference between the satisfaction scores of preclinical and clinical students in favor of preclinical students. The results showed that there were significant differences between preclinical and clinical students with regard to the strengths of SBL in favor of preclinical students. They reported that exposure to the semi-realistic environment provided them with real patient-related information, enhanced their communication skills, and motivated their learning.

To sum up, it can be clearly noted from the results that, within the framework of experiential learning, increasing simulator use in teaching and adding more exercises might contribute to better learning outcomes.

Limitations

Although the findings of the current study indicate satisfaction with SBL, there are some limitations to this study that may prevent the generalization of these findings. One of these limitations is that the study was conducted at one institution (KSU). It would have been better to include more than one institution. Moreover, there was no control group that would have allowed a comparison of the effects of other teaching modes on students’ learning. Regardless of these limitations, the findings of this study can guide stakeholders’ understanding of how students’ learning needs should be addressed. It is also recommended that future studies include larger samples and use mixed approaches.

## Conclusions

Simulation is a well-accepted format for incorporating experiential learning into medical institutions’ curricula. SBL provides learners with a safe and controlled environment. This study’s results showed high satisfaction levels with using SBL as a method for clinical teaching. SBL was recognized as a useful and effective way of learning skills, and students reported that it helped them to implement what they had learned. However, lack of accessibility and lack of trained staff were reported, and these issues should be addressed by providing staff with proper training. Hence, providing simulation-based training is a pathway compliant with the best educational standards that should be adapted according to each institution’s unique needs and circumstances.
